# Ergogenic effects of betaine supplementation on strength and power performance

**DOI:** 10.1186/1550-2783-7-27

**Published:** 2010-07-19

**Authors:** Elaine C Lee, Carl M Maresh, William J Kraemer, Linda M Yamamoto, Disa L Hatfield, Brooke L Bailey, Lawrence E Armstrong, Jeff S Volek, Brendon P McDermott, Stuart AS Craig

**Affiliations:** 1 Department of Kinesiology, University of Connecticut, Storrs, CT, USA; 2 Danisco A/S, Tarrytown, NY, USA

## Abstract

**Background:**

We investigated the ergogenic effects of betaine (B) supplementation on strength and power performance.

**Methods:**

Twelve men (mean ± SD age, 21 ± 3 yr; mass, 79.1 ± 10.7 kg) with a minimum of 3 months resistance training completed two 14-day experimental trials separated by a 14-day washout period, in a balanced, randomized, double-blind, repeated measures, crossover design. Prior to and following 14 days of twice daily B or placebo (P) supplementation, subjects completed two consecutive days (D1 and D2) of a standardized high intensity strength/power resistance exercise challenge (REC). Performance included bench, squat, and jump tests.

**Results:**

Following 14-days of B supplementation, D1 and D2 bench throw power (1779 ± 90 and 1788 ± 34 W, respectively) and isometric bench press force (2922 ± 297 and 2503 ± 28 N, respectively) were increased (p < 0.05) during REC compared to pre-supplementation values (1534 ± 30 and 1498 ± 29 W, respectively; 2345 ± 64 and 2423 ± 84 N, respectively) and corresponding P values (1374 ± 128 and 1523 ± 39 W; 2175 ± 92 and 2128 ± 56 N, respectively). Compared to pre-supplementation, vertical jump power and isometric squat force increased (p < 0.05) on D1 and D2 following B supplementation. However, there were no differences in jump squat power or the number of bench press or squat repetitions.

**Conclusion:**

B supplementation increased power, force and maintenance of these measures in selected performance measures, and these were more apparent in the smaller upper-body muscle groups.

## Background

As an organic osmoprotectant and source of methyl groups betaine is involved in diverse cytoprotective and metabolically beneficial pathways in plants, animals, and prokaryotes [1,2]. Recent human research has also examined the ergogenic potential of betaine in endurance and resistance exercise [3-6].

Armstrong et al. [3] reported non-significant trends (21% and 16%) toward longer sprint duration performed at 84% VO_2 _max to volitional exhaustion in male runners following acute ingestion of 5 g betaine combined with water or a carbohydrate-electrolyte fluid, respectively, compared to corresponding control trials. In the only study published to date on the effects of prolonged (14-15 days) betaine supplementation (1.25 g twice per day) on power performance, Hoffman and coworkers [6] reported no significant differences between betaine and placebo groups in the total repetitions performed to exhaustion at 75% 1RM, or in the number of repetitions performed at 90% of both peak and mean power, in the bench press exercise. However, the number of repetitions performed in the squat exercise was greater (p < 0.05) on days 7-8 of betaine ingestion, and showed a similar trend (p = 0.06) on day 14-15, compared to the placebo group. There were no differences between groups in vertical jump power, in bench press throw power, or in the Wingate anaerobic power test.

Though little is yet known about the mechanisms, there is some evidence that betaine supplementation may positively affect exercise performance through favorable lactate and preferential fatty acid substrate metabolism [3,5]. Additionally, betaine may be involved in defending intracellular volume [7,8] and protecting enzymes of the citric acid cycle [2], which are challenged in progressive dehydration and hyperthermia associated with exercise. Less definitively, betaine's relationship to choline, methionine, serine, vitamin B metabolism, and methyl donating reactions may all contribute to its ergogenic efficacy [2].

Considering the known importance of dietary betaine, the safety of betaine supplementation [2], and prevalence of betaine in foods typical of affluent American diets [9], this study aimed to further investigate the yet undefined ergogenic effects of betaine on resistance exercise, particularly on strength and power performance. To this end, we conducted a carefully controlled randomized crossover design study using recreationally active men with at least three months of resistance training experience. We hypothesized that betaine supplementation would be associated with improved strength and power in these individuals, thus demonstrating the potential efficacy of betaine in improving performance and recovery in strength and power exercise.

## Methods

### Subjects

Twelve healthy, recreationally active men (mean ± SD age, 21 ± 3 yr; mass, 79.1 ± 10.7 kg) participated. A within-treatment experimental design was used to increase sensitivity and reliability of measures and thus, each subject acted as his own control. Subjects were matched according to age, body size, and training experience prior to their initial random placements into one of the two treatment conditions. Eligibility required at least three months of resistance training experience including the squat exercise. Medical histories were obtained to exclude medical, musculoskeletal, and endocrine disorders, concurrent nutritional supplementation, and anabolic drugs. All subjects were informed of the benefits and potential risks of the investigation and signed a University Institutional Review Board approved consent form for recruitment and participation.

### Study design

A balanced, randomized, double blind, repeated-measures, placebo, cross-over design was used. All subjects performed a testing protocol providing direct data on physical performance. Recovery effects were measured by repeating this testing protocol 24 hr following this first visit. After this initial (baseline) testing, subjects underwent 14 days of betaine or placebo supplementation again followed by exercise testing on two consecutive days. Subjects underwent a 14 day washout period and then crossed over into the other 14-day period of either betaine or placebo supplementation. In addition to performance testing, some blood variables were measured, and special attention was given to dietary and activity control among and within subjects. Subjects refrained from any exercise for 48 hr prior to the scheduled performance testing sessions. All testing sessions were conducted between 0700 and 1000 hr, but at the same time of day for each respective subject. A standardized whole-body resistance training session was performed twice (mid-week) during the 14-day supplementation periods to maintain the subjects' level of conditioning.

### Betaine supplementation

Betaine supplement (B) was given as 1.25 grams (g) of betaine (Danisco Inc., Ardsley, NY) in 300 mL of Gatorade^© ^sports drink, taken twice daily at standardized times for each subject. Additionally, on each testing day subjects received a morning dose of the betaine supplement or placebo. Placebo (P) drinks were the same sports drink formulation and flavor without the betaine additive. Researchers involved in data collection and participants themselves were blinded to treatment until an un-blinded outside researcher revealed treatments following study completion.

### Exercise testing protocol

After a standardized warm up of 5 minutes of low intensity cycling, subjects performed the following high intensity strength/power resistance exercise challenge (REC).

4 sets × 3 repetitions Vertical Jump

2-minute rest following each set

Maximal effort Isometric Squat (lasting 6-10 sec)

2-minute rest

3 repetitions Squat Jump @ 30% 1 RM

2-minute rest

3 sets Back Squat @ 85% 1 RM until fatigue

2-minute rest following each set

Maximal effort Isometric Bench Press (lasting 6-10 sec)

2-minute rest

3 repetitions Bench Throw @ 30% 1 RM

2-minute rest

3 sets Bench Press @ 85% 1 RM until fatigue

2-minute rest following each set

Standardized resistance exercise testing protocols are commonly used in our laboratory for research studies [e.g. [10,11]]. During this protocol, measures of power (W) and force (N) were measured using a force plate (AccuPower, Athletic Republic, Fargo, ND, USA).

### Blood variables

Blood samples were collected via an indwelling catheter placed in the antecubital forearm vein at the beginning of each day of exercise testing. Samples were obtained before exercise testing began, immediately following vertical jump, following squat testing, immediately post all exercise testing, and fifteen minutes following cessation of exercise, for a total of five blood timepoints. After whole blood analyses, blood plasma was obtained via centrifugation (Hettich Centrifuge, Beverly, MA) at 3200 RPM, 4°C, 20 minutes, and stored at -80°C until further analysis. Betaine was analyzed in EDTA preserved plasma samples. High performance liquid chromatography was utilized with a silica column in a mixed partition and ion exchange mode following a method previously described [12]. Hematocrit (International Equipment Co., Needham Heights, MA, microcapillary reader) and hemoglobin concentration (Hemocue 201+ Analyzer, Lake Forest, CA) were obtained from whole blood, plasma osmolality was measured with an osmometer (Advanced Instruments, Inc., Norwood, MA, Model 3250) prior to sample storage. Glucose and lactate concentrations were analyzed using a glucose/lactate analyzer (2300 YSI Stat Plus, Yellow Springs, OH). All blood variables were measured in respective SI units.

### Other variables

Subjects submitted self-administered 3-day diet records and six week activity records to verify consistency in diet and activity during study participation. Urine specific gravity (USG) (ATAGO clinical refractometer, Cole-Parmer, Vernon Hills, IL), osmolality, and body mass were measured prior to each exercise testing session to verify hydration status.

### Statistical analysis

All variables were analyzed using Repeated Measures ANOVA with supplement treatment (placebo or betaine, two levels) and the appropriate number of time points as within subject factors. The sphericity assumption was met and significance was set at p < 0.05. Post hoc comparisons were t tests with Bonferroni corrections applied. The main effects of supplement were evaluated in the statistical model, and time effect and supplement × time interaction effects were also evaluated. Data are presented as means ± standard deviation for all variables.

## Results

Subjects reported that they could not distinguish which treatment (P or B) they received in either of the two phases of supplementation. All subjects reported similar physical activity and diet prior to each exercise test and throughout study participation. Subjects exhibited no significant change in weight over the course of the study, or between treatment periods (P Pre = 80.1 ± 10.5 kg, B Pre = 80.2 ± 11.5 kg, P Post = 80.3 ± 11.8 kg, B Post = 80.6 ± 11.3 kg).

Additionally, prior to each treatment phase, subjects exhibited no differences in hydration state determined by measures of urine specific gravity, averaging 1.019 ± .008 pre-testing during D1 and D2 for both the P and B conditions [13].

After 14 days of B supplementation, plasma betaine concentrations were significantly greater than corresponding baseline and placebo (48 ± 10 μmol/L) levels.

There were no differences in power output measures (W) for the four vertical jumps performed on D1 or Day 2 before P or B supplementation, or after 14 days of P supplementation. However, following the 14 days of B supplementation there were significant increases in power output for two of these four vertical jumps performed on D1 (4980 ± 61 and 5085 ± 137 W, respectively) and D2 (4811 ± 77 and 5068 ± 529 W, respectively) compared to corresponding D1 (4545 ± 114 and 4452 ± 130 W, respectively) and D2 (4476 ± 96 and 4848 ± 91 W, respectively) pre-supplement values.

**Figure 1:**
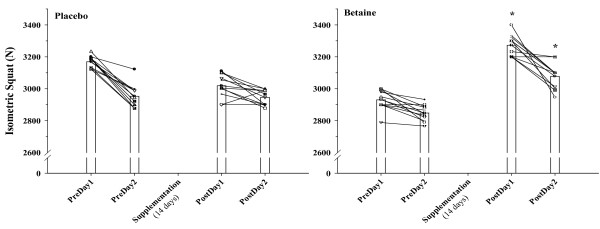
**Individual (n = 12) and mean responses for squat jump power (W, Watts) on the two days before (PreDay) and after (PostDay, 14 days) placebo and betaine supplementation**. * = p < 0.05 from corresponding betaine PreDay value.

Subjects exhibited decreased or similar force production in the isometric squat before and after P, but this was significantly improved on D1 and D2 after 14 days of B supplementation compared to pre supplement measures. Figure 1 illustrates these differences.

**Figure 2:**
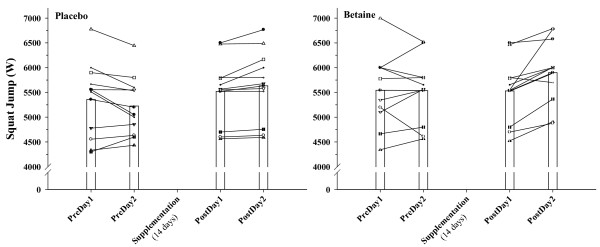
**Individual (n = 12) and mean responses for squat jump power (W, Watts) on the two days before (PreDay) and after (PostDay, 14 days) placebo and betaine supplementation**.

Squat jump power was not significantly different between P and B, nor was it different from pre- to post- testing for either treatment. There was also greater sample variation among individuals with respect to this test as can be seen in Figure 2.

As shown in Table 1 there were no significant differences between the P and B trials in the total number of back squat repetitions performed at 85% of 1 RM until fatigue.

**Table 1:** Total number of repetitions to fatigue in the back squat during the two days before and after supplementation (n = 12)

	Placebo	Betaine
Pre-Testing	16 ± 1	16 ± 2
Day 1		
		
Pre-Testing	14 ± 2	14 ± 2
Day 2		
		
Post-Testing	15 ± 2	16 ± 2
Day 1		
		
Post-Testing	14 ± 2	16 ± 2
Day 2		

**Figure 3:**
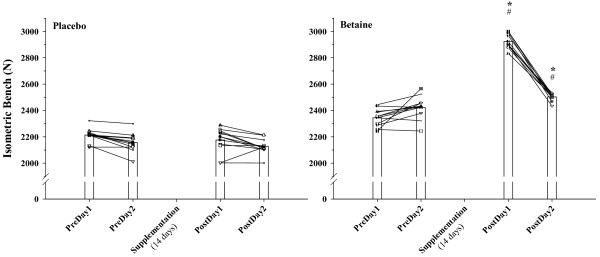
**Individual (n = 12) and responses for isometric bench force (N, Newtons) on the two days before (PreDay) and after (PostDay, 14 days) placebo and betaine supplementation**. * = p < 0.05 from corresponding betaine PreDay value, # = p < 0.05 from corresponding placebo PostDay value.

Figure 3 shows improvements in isometric bench force following B supplementation. This B versus P difference was approximately 800 N greater on D1 and approximately 400 N greater on D2.

**Figure 4:**
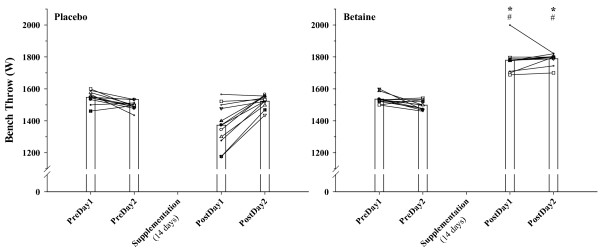
**Individual (n = 12) and mean responses for bench throw power (W, Watts) on the two days before (PreDay) and after (PostDay, 14 days) placebo and betaine supplementation**. * = p < 0.05 from corresponding betaine PreDay value, # = p < 0.05 from corresponding placebo PostDay value.

Figure 4 illustrates that bench throw power also significantly improved following 14 days of B supplementation on both D1 and D2 testing.

Similar to the back squat, there were no significant differences between the P and B trials in the total number of bench press repetitions performed at 85% of 1 RM until fatigue. These values are presented in Table 2.

**Table 2:** Total number of repetitions to fatigue in the bench press during the two days before and after supplementation (n = 12)

	Placebo	Betaine
Pre-Testing	12 ± 1	10 ± 1
Day 1		
		
Pre-Testing	12 ± 2	12 ± 1
Day 2		
		
Post-Testing	13 ± 1	11 ± 1
Day 1		
		
Post-Testing	13 ± 1	11 ± 1
Day 2		

Hematocrit (%), hemoglobin (g/dL), and plasma osmolality (mOsm/kg) were significantly greater at post-squat (49 ± 1, 15.7 ± 1.0, 303 ± 4, respectively) and immediately after REC (48 ± 1, 16.0 ± 1.0, 303 ± 3, respectively) compared to pre-exercise values (43 ± 1, 14.3 ± 0.8, 289 ± 3, respectively) during D1 and D2 testing, but these values were not significantly different between the P and B trials.

Plasma glucose was not different before P or B supplementation (5.1 ± 0.6 and 5.0 ± 0.7 mmol/L, respectively) or at any time in response to the REC protocol (averaging 5.1 ± 0.5 and 5.1 ± 0.8 mmol/L, respectively) after P or B supplementation. As expected, plasma lactate showed significant increases above average pre exercise (1.4 ± 0.4 mmol/L) values throughout the REC protocol on both D1 and D2 testing days, and this increase (8.7 ± 2.2 and 8.8 ± 1.8 mmol/L, respectively) was the same for P and B exercise testing sessions.

## Discussion

There is an increased interest in the study of betaine as an ergogenic supplement for the neuromuscular system. In the current study, the primary effect of the betaine supplement was observed in the upper body, with enhanced bench press force and power production, but no change in the dynamic squat exercise performances. Additionally, the improvements in the bench press performances were observed on D2, demonstrating the efficacy of betaine as a potential aid to recovery. This is in contrast to the recent findings by Hoffman et al. [6] who demonstrated improvements in squat exercise endurance (i.e., number of repetitions to failure at 90% of the 1 RM yet not at 75% of the 1RM), but no changes in these measures in the bench press or for the lower body Wingate test. This disparity in results is likely due to a host of differences in the study design and dependent variables. Firstly, we utilized a within versus between group experimental design allowing greater control of statistical variance. Secondly, our study employed a different sequence of exercises and repetitions and our primary dependent variables were the peak force and power, rather than the force and power specific to local muscular endurance defined by the number of repetitions to failure at 75% and 90% of the 1RM. However, we did find that high force production improved with betaine supplementation which reflects some similarity to the study by Hoffman and coworkers. While the muscle groups in the two studies were apparently different in their mediating mechanisms, both studies provide evidence for the potential positive influence of B supplementation for strength, power and local muscular endurance in the context of demanding strength/power exercise protocols.

In the present study, the larger lower-body muscle group data was more varied within the subject sample and significant differences were less obvious, although patterns of B mediated increases may be suggested. For example, isometric squat force was enhanced by B supplementation. The REC protocol utilized maximal vertical jumps prior to the squat exercises which might have impaired the neuromuscular performance of high power production as recently noted by Drinkwater et al. [14], indicating that order of exercises is an important element in training program design. In this case, the betaine supplement was likely not able to offset the neural effect and partially explains the lack of improved power production in the squat. However, force production may have been facilitated via a post activation potentiation effect of some type [15]. While speculative, the upper body musculature was not inhibited by such an inhibitory neuromuscular influence of high velocity power movements as was the lower body in this exercise testing sequence. Thus, it appears that the mediating mechanisms of betaine supplementation may be more operational in the absence of high frequency neural fatigue.

From the non-significant differences in body fluid related variables between the B and P trials, due to the experimental controls for hydration employed in this study, it seems that betaine's established role as an osmoprotectant [2,7,8] was not a likely candidate for any ergogenicity. This does not, however, minimize the potential role of betaine given the intensity of the REC, as organic osmolytes have been shown to accumulate in cells under varying stressful conditions to help maintain biochemical function [16-18]. Additionally, plasma glucose and lactate results in this study indicate that betaine was either 1) not acting through glucose or lactate processing, or 2) the pre-existing differences among subjects masked any betaine effects on these dependent variables. The use of the very demanding REC might have overwhelmed the ability of betaine to offer any measureable differences, which in the case of the enhanced performances would most likely be related to phosphagen metabolism. Furthermore, the link between betaine as a methyl donor and improved exercise performance can only be speculated to be related to such variables as methionine, choline, and creatine [5,19-23]. The contribution of betaine to these specific relationships should be examined in future studies.

## Conclusions

Betaine has been shown to have numerous, diverse, positive effects [2] and in the current study betaine supplementation corresponded positively with gains in bench throw power, isometric bench press force, some measures of vertical jump power, and isometric squat force. However, precise mechanistic inferences will require further direct investigation while accounting for neural inhibitory factors. Considering the previous results from our laboratory demonstrating the effect of betaine on high intensity exercise performance in hot environments [3], and those recently reported by Hoffman et al. [6] on the quality of power test repetitions and endurance during power tests, it seems that betaine ergogenicity merits further research in both endurance and strength/resistance exercise.

## Competing interests

The first nine authors, all associated with the University of Connecticut at the time of this study, declare that they have no competing interests. SASC is employed by Danisco A/S, the company that funded this study. Publication of these findings should not be viewed as endorsement by the investigators, the University of Connecticut, or the editorial board of the Journal of the International Society of Sport Nutrition.

## Authors' contributions

CMM was the primary investigator, obtained grant funds for the project, and supervised all study recruitment, data acquisition, data specimen analysis, and manuscript preparation. CMM and WJK designed the study protocol. ECL, LMY, DLH, BLB, and BPM made substantial contributions to data acquisition. LEA and JSV made substantial contributions to interpretation of data. ECL performed the statistical analysis and was primarily responsible for writing the manuscript. CMM, WJK, LMY and SASC were also involved in manuscript writing and preparation.

All authors have read and approved the final manuscript.
